# TAGOPSIN: collating taxa-specific gene and protein functional and structural information

**DOI:** 10.1186/s12859-021-04429-5

**Published:** 2021-10-23

**Authors:** Eshan Bundhoo, Anisah W. Ghoorah, Yasmina Jaufeerally-Fakim

**Affiliations:** 1 Department of Agricultural and Food Science, Faculty of Agriculture, University of Mauritius, Reduit, 80837 Mauritius; 2 Department of Digital Technologies, Faculty of Information, Communication and Digital Technologies, University of Mauritius, Reduit, 80837 Mauritius

**Keywords:** Comparative genomics, Data integration, Data retrieval, Database, Object-oriented biology

## Abstract

**Background:**

The wealth of biological information available nowadays in public databases has triggered an unprecedented rise in multi-database search and data retrieval for obtaining detailed information about key functional and structural entities. This concerns investigations ranging from gene or genome analysis to protein structural analysis. However, the retrieval of interconnected data from a number of different databases is very often done repeatedly in an unsystematic way.

**Results:**

Here, we present TAxonomy, Gene, Ontology, Protein, Structure INtegrated (TAGOPSIN), a command line program written in Java for rapid and systematic retrieval of select data from seven of the most popular public biological databases relevant to comparative genomics and protein structure studies. The program allows a user to retrieve organism-centred data and assemble them in a single data warehouse which constitutes a useful resource for several biological applications. TAGOPSIN was tested with a number of organisms encompassing eukaryotes, prokaryotes and viruses. For example, it successfully integrated data for about 17,000 UniProt entries of *Homo sapiens* and 21 UniProt entries of human coronavirus.

**Conclusion:**

TAGOPSIN demonstrates efficient data integration whereby manipulation of interconnected data is more convenient than doing multi-database queries. The program facilitates for instance interspecific comparative analyses of protein-coding genes in a molecular evolutionary study, or identification of taxa-specific protein domains and three-dimensional structures. TAGOPSIN is available as a JAR file at https://github.com/ebundhoo/TAGOPSIN and is released under the GNU General Public License.

**Supplementary Information:**

The online version contains supplementary material available at 10.1186/s12859-021-04429-5.

## Background

With advances in experimental techniques, biological data are now readily available in public databases and are becoming even more so at an ever-increasing pace. As an illustration, the popular NCBI GenBank [1] stores nucleotide sequence data, UniProt Knowledgebase (UniProtKB) [2] stores protein data, the RCSB PDB [3] stores 3D structural data, and Pfam [4] gathers information about protein domain families. Although these databases have greatly simplified the task of researchers needing select, formatted data for a particular investigation, retrieving the required data is too often done repeatedly in an unsystematic way. Yet, those databases represent valuable resources to tackle studies in genome evolution [5, 6] and comparative genomics [7], drug development [8, 9], analysis of protein domains [10, 11] and search of new motif-binding domains [12]. All of these studies involve a data preparation step, which requires querying multiple databases, cross-referencing, removing duplicates and missing data, and filtering out any inconsistencies in order to obtain a clean data set for the analysis stage. Despite being common to the above-mentioned studies, the data preparation step is still being done on an individual basis. Consequently, similar research studies by different groups cannot be compared because their data set varies, enormous efforts are spent on data preparation, and no comprehensive data set is used. Hence, there is a need for a systematic way of retrieving and integrating public information available for a given organism.

Currently, NCBI Entrez Programming Utilities (E-utilities) [13], UniProtKB and EBI SIFTS [14] are among the most widely used data retrieval and cross-referencing tools. The E-utilities serve as the Application Programming Interface (API) for the Entrez system [15] which is a query and database retrieval system giving access to 38 databases that together contain 2.5 billion records. UniProtKB is the nucleus of protein knowledge, offering scientists extensive and high-quality information about protein sequences and annotations. Most of these protein sequences are translations of the coding sequences submitted to the public nucleic acid databases, which include NCBI GenBank. EBI SIFTS for its part aims to integrate structural data in the PDB [16] with sequence data in UniProtKB by providing up-to-date residue-level mapping between protein structure and amino acid sequence. It also provides up-to-date residue-level cross-references between PDB structures and data available in several other biological databases, among which Gene Ontology (GO) [17] and Pfam. However, even though the three resources are invaluable in many research studies, including the aforementioned ones [5–12], they do not provide a way of concomitantly retrieving and integrating data from multiple databases into a single repository for further analyses. To the best of our knowledge there is no such tool.

Using an object-oriented approach to biology [18] in which organism, genome, gene, protein, biological function, protein domain family and protein 3D structure are conveniently modelled as real-world entities, we developed TAGOPSIN, a command line program which retrieves and organises useful data for each entity. Besides other practical applications, TAGOPSIN facilitates on one hand selection of taxa-specific nucleotide or amino acid sequences to investigate for example interspecific evolutionary events [19], and on the other hand search of a novel protein drug target for docking-based virtual screening (DBVS). Indeed, DBVS has shown great promise for the identification of novel therapeutic leads [20].

## Implementation

### General description

TAGOPSIN is written in Java 8. It is the acronym for Taxonomy, Gene, Ontology, Protein, Structure Integrated. It prompts a user for the name of an organism of interest, and accordingly retrieves select data from seven biological repositories, namely NCBI Taxonomy [21], NCBI RefSeq [22], Gene Ontology [17], UniProtKB/Swiss-Prot [2], Pfam [4], EBI SIFTS [14] and RCSB PDB [3]. The organism can be either a eukaryote, a prokaryote or a virus. Fed into a centralised local relational database managed by PostgreSQL, the retrieved data can then be utilised on a user-defined basis.

### System requirements

TAGOPSIN is designed for Unix-based operating systems and requires the relational database management system PostgreSQL. It was tested on a Linux Ubuntu system (version 18.04) with PostgreSQL 10.

### Methods

**Fig. 1:**
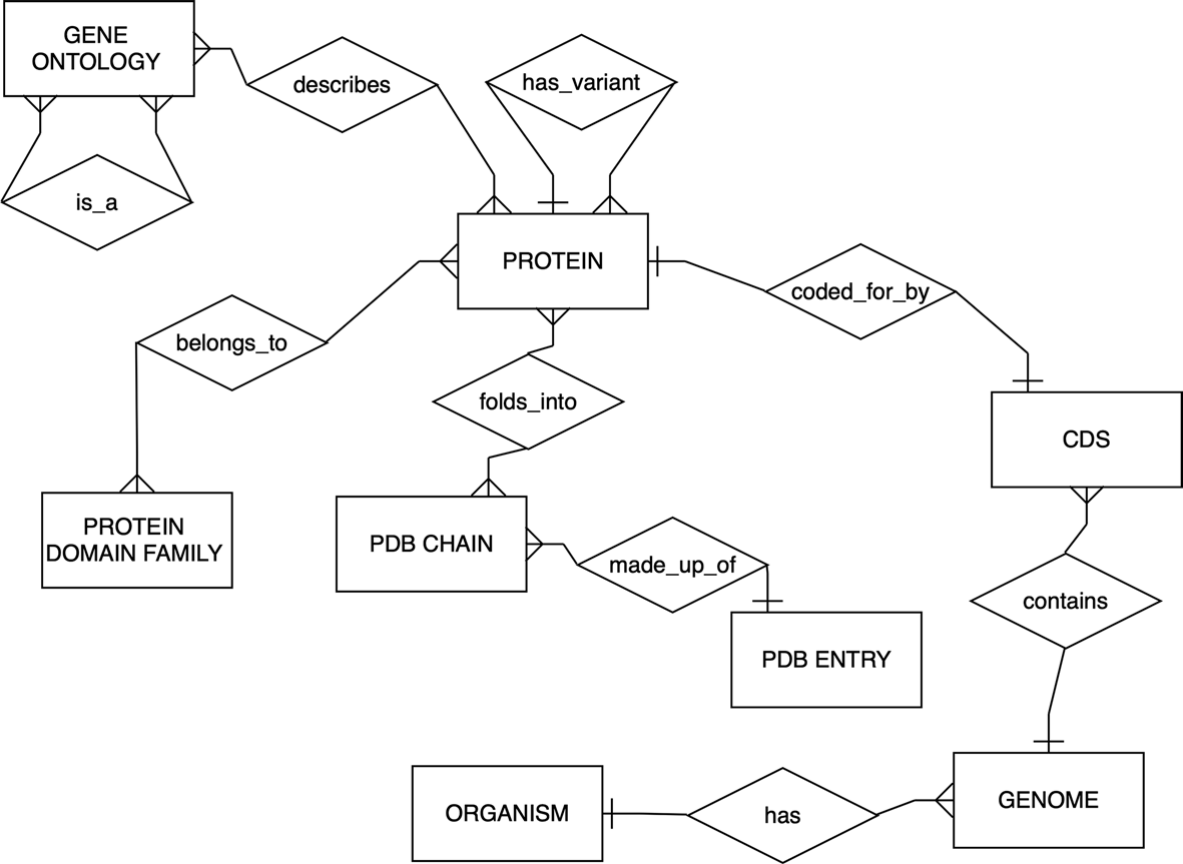
**The data model of TAGOPSIN,** represented as an ERD

In database terminology, an entity is a real-world object with independent existence and an attribute is a characteristic describing the entity. Organism, genome, coding sequence (CDS), protein, biological function (ontology), protein domain family and protein 3D structure are modelled as entities. The interrelationship between the different entities can be represented schematically in an entity-relationship diagram (ERD) (Fig. 1).

**Fig. 2:**
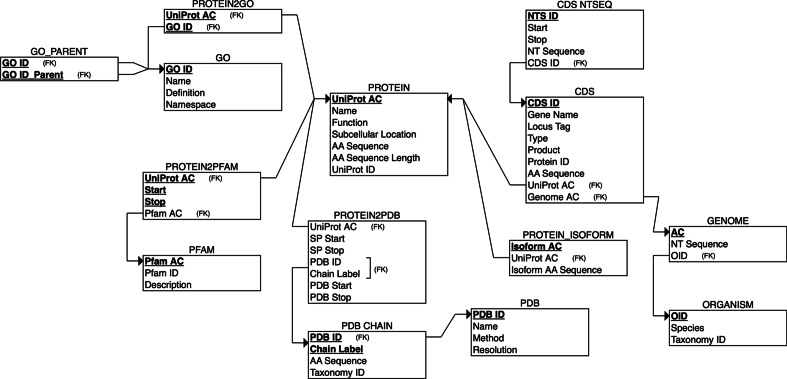
**Relational database schema** representing schematically the data warehouse built by TAGOPSIN. Primary key (PK) attributes (allowing unique identification) are in bold and underlined. An arbitrary PK was chosen where a satisfactory biological attribute could not symbolize it (e.g. *OID*, *CDS ID*, *NTS ID*). Foreign key attributes are indicated by *(FK)*. Arrows indicate how one relation is linked to another. *Start, Stop*: start and end positions on the corresponding sequence; *SP Start, SP Stop*: start and end positions on the Swiss-Prot sequence; *PDB Start, PDB Stop*: start and end positions on the PDB structure

**Fig. 3:**
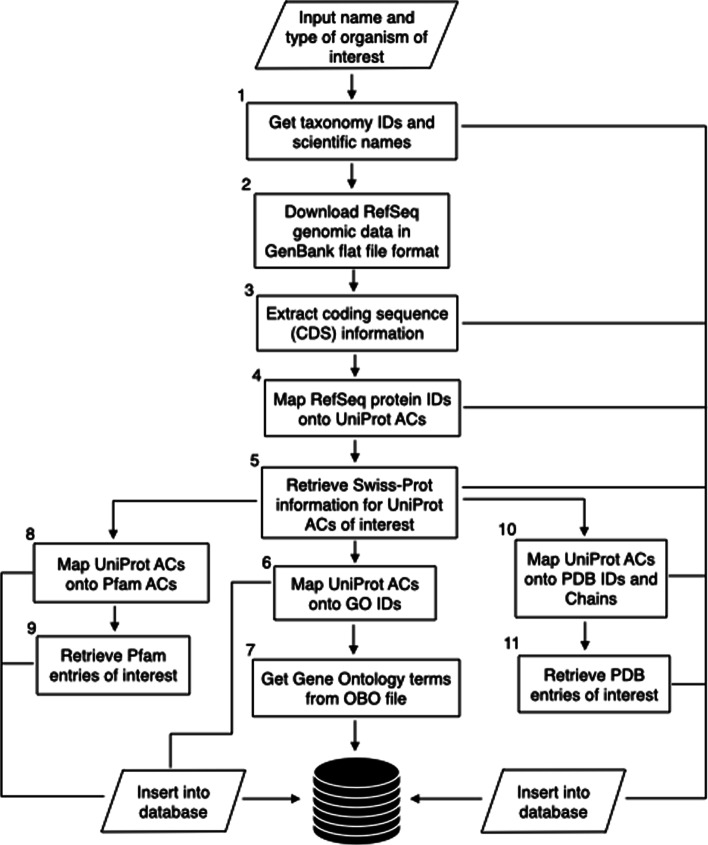
Sequential steps in the functioning of TAGOPSIN

For each entity, raw data files are downloaded from their respective FTP or HTTP servers, namely NCBI Taxonomy, NCBI Nucleotide, UniProtKB, Gene Ontology, Pfam and EBI SIFTS / RCSB PDB. RefSeq data in GenBank flat file format are retrieved from NCBI Nucleotide [13] via E-utilities, using shell scripting and the Unix command wget. TAGOPSIN parses each data file and extracts information relevant to the different attributes of each entity (Fig. 2). Shell scripts split the genome into sequence bins so as to facilitate extraction of the CDSs. Additionally, files containing database cross-references are also downloaded and parsed. Once taxonomy IDs of the organisms of interest are known, data retrieval is restricted to only those IDs in subsequent data files, as illustrated in Fig. 3. All information retrieved is inserted directly into a relational database in PostgreSQL (Fig. 3), as per an established relational database schema (Fig. 2).

## Results

We present a tool that integrates multiple biological databases into one unified repository. Coupled with basic knowledge of structured query language (SQL), manipulation of interconnected data is more convenient than doing multi-database queries. Importantly, several biological applications are envisaged. In particular, the data retrieved can be efficiently used in interspecific comparative analyses of protein-coding nucleotide sequences to study molecular evolution. Moreover, a study of genes and/or proteins by biological process or molecular function is feasible for a given organism. The identification of taxa-specific protein domains and/or 3D structures for investigations requiring such entities (e.g. DBVS) is also facilitated.

We tested the functionality of TAGOPSIN with seven organisms representative of eukaryotes, prokaryotes and viruses. The program was queried using the genus or species name. Table 1 lists the statistics of the dataset retrieved by TAGOPSIN for each one of these organisms, classified by entity type.

**Table 1:** Case study of the performance of TAGOPSIN

Query	*H. sapiens*	*A. thaliana*	*S. cerevisiae*	*E. coli*	*Streptococcus*	Human coronavirus	Human papillomavirus
No of organisms							
From Taxonomy	7	5	351	3 365	5 039	27	259
From Nucleotide	0	0	0	1 142	637	2	33
No of curated genomes/chromosomes	24	5	16	1 274	796	4	65
No of CDSs	112 702	48 147	5 989	6 066 761	1 540 276	30	442
No of proteins	17 261	15 383	5 876	17 777	9 037	21	127
No of protein isoforms	16 007	2 196	29	6	0	0	0
No of GO terms	17 597	6 825	6 023	2 614	815	47	54
No of protein domain families	6 108	3 125	3 222	2 281	819	36	12
No of protein 3D structures	39 885	1 472	4 565	1 893	232	27	36
Approx. runtime (hours)	147.83	5.31	3.03	72.35	19.52	3.01	3.18

The statistics of the datasets built for *Homo sapiens*, *Escherichia coli* and five other organisms, as indicated, is classified by entity type. Estimated runtimes are on a 64-bit Linux Ubuntu system with 4.7 GiB of RAM and an Intel$^{\circledR }$ Core^TM^ i7-6500U CPU @ 2.50 GHz processor. Actual runtimes may vary depending on Internet bandwidth, volume of data to process, and hardware specifications. Here an average bandwidth of 1.1 MB/s was used. The times indicated for *H. sapiens* and *E. coli* include time to download and decompress standard data files (updated July/August 2020)

With 4.7 GiB of RAM and an Intel$^{\circledR }$ Core^TM^ i7-6500U CPU @ 2.50 GHz processor, TAGOPSIN showed satisfactory performance to build the datasets in Table 1. The runtime is dependent on the size and complexity of the genome, the volume of data to process and a user’s bandwidth (here average bandwidth 1.1 MB/s). Overall, the program took less than 24 hours for organisms with a relatively small genome (e.g. *A. thaliana*, *S. cerevisiae*), approximately 72 hours to build the dataset for *Escherichia coli* (1,274 genomes), and 148 hours for *Homo sapiens* (24 chromosomes). TAGOPSIN also retrieves available UniProt ACs and amino acid sequences of protein isoforms. In our case study of its performance, as expected TAGOPSIN obtains significant data on protein isoforms for all three eukaryotes (Table 1). This information could help a user to conveniently probe sequence variations and their effects.

**Fig. 4:**
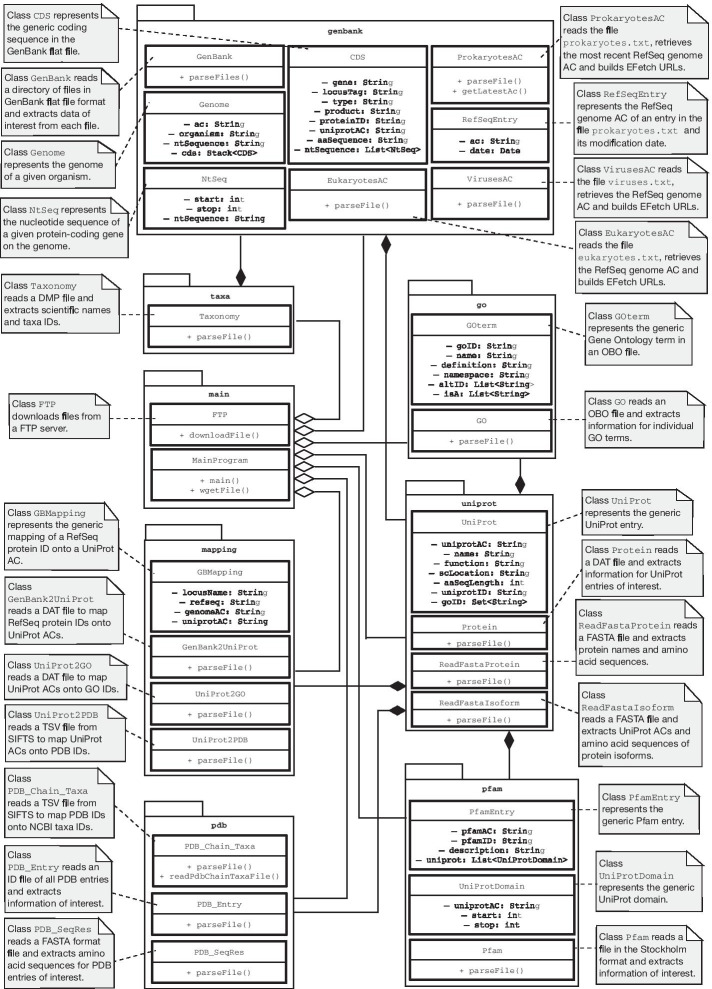
Class diagram illustrating the data model of TAGOPSIN

A unique feature of TAGOPSIN is its object-oriented approach to biology whereby biological entities are represented as Java classes and objects. We depict this feature in a class diagram (Fig. 4). Class diagrams have been used earlier to model processes in molecular biology, for example protein-protein interactions (PPIs) [18]. Here we use the same representation to model the flow of information from DNA to protein, and subsequently protein structure and function. Furthermore, using modular programming and the object-oriented paradigm, the functionality of TAGOPSIN is separated into independent modules as shown in Fig. 4. The module *go* for example allows the retrieval of biological functions for the proteome of a specific organism. Likewise, the module *genbank* allows the retrieval of that organism’s entire set of protein-coding genes. This object-oriented nature of TAGOPSIN also enables easy reusability and maintenance of code.

While functionally similar tools like E-utilities, UniProtKB and EBI SIFTS cross-reference useful biological information and/or allow its retrieval, to the best of our knowledge they do not provide a way of simultaneously retrieving and collating interconnected data in a single repository for ease of data manipulation. Here, we propose a command line program that retrieves and consolidates data for a specific organism from seven biological resources widely used in comparative genomics and structure-function relationship studies. TAGOPSIN includes taxonomy details and thus limits data retrieval to only a particular organism of interest (Fig. 3).

## Discussion

### Benchmarking TAGOPSIN

The data warehousing strategy provides one single access point to conveniently manipulate a wide range of data from disparate sources. Most of the biological data warehouses reported previously in the literature are now obsolete [23–26]. There exists however BioWarehouse [27] and JBioWH [28] which are accessible and which have the characteristic of integrating various databases into one. In this section, we compare the functionality and performance of TAGOPSIN with these two resources as well as E-utilities, UniProtKB, EBI SIFTS, and the two graph-based data platforms Bio4j [29] and Neo4j [30]. We also benchmark TAGOPSIN against the bioDBnet web server^1^ and the WikiGenomes web application [31].

*BioWarehouse* BioWarehouse constructs data warehouses by combining different bioinformatics databases within a single database management system to facilitate multi-database queries using SQL [27]. Although the home page indicates that BioWarehouse services have shut down since 2015, the source code is still available for download and web-based search functions of the BioCyc collection are operational. We thus endeavoured to assess the output of TAGOPSIN relative to that of BioCyc resources. Amongst these, MetaCyc was queried for all gene products of the genus *Streptococcus*. It returned 85 products for 9 species. Yet, these products are specific to metabolic pathways and enzymes, and the result set does not include for instance coding sequence data or 3D structural data. By contrast, TAGOPSIN retrieves the entire set of gene products along with their annotations and sequences for each organism, and here, it returns over 1 m coding genes for 52 species of *Streptococcus* (Additional file 1), but also all available data on gene ontology and protein 3D structure (Table 1).

Another BioCyc resource is EcoCyc which, like TAGOPSIN, provides cross-references to structure and sequence databases. Nevertheless, it is specific to *E. coli* strain K-12 substr. MG1655. Biological information for other strains of *E. coli*, which can be invaluable in the context of comparative genomics, is thus not available in one and the same repository. Similarly, EcoliHouse^2^ was constructed using BioWarehouse 4.6 but collates data for *E. coli* K-12 only.

In general, databases of the BioCyc collection, which include MetaCyc and EcoCyc, comprise many features and tools. Despite the fact that not all of them are readily available (some cannot be used without a subscription), an interesting feature is the possibility to visualise, manipulate and analyse omics data (e.g. transcriptomics, proteomics, metabolomics). At present, TAGOPSIN does not take into account such type of biological information since our primary motivation was to develop a program that would help in comparative genomics. Secondly, on the ‘Gene Search’ result page, even though the tool ‘Search for This Gene in Multiple Databases’ under ‘Comparison Operations’ outputs that same gene or its orthologs in different strains of a species, we do note however that the accessions in the result set are not RefSeq accessions. If the latter are provided, they are on individual result pages and hyperlinked to the record in the parent database. One of the main strengths of TAGOPSIN is that it collects only the RefSeq subset of GenBank to produce a non-redundant CDS dataset which is important when comparing organisms in a clade.

It should be noted that the ‘Comparative Analysis’ tool of each member database of the BioCyc collection does not allow comparison of more than 70 organisms at a time. Using this tool on MetaCyc.org website, we tried to compare 20 random *Streptococcus* species and it resulted in statistics in the form of numbers of genes, proteins and other gene products. However, a similar query on EcoCyc.org website with 35 random *E. coli* strains failed due to gateway timeout.

*JBioWH* The data warehouse JBioWH has 20 component databases. While TAGOPSIN works with complex relational data, JBioWH implements a graph data structure which is appropriate for network representation of biological entities. This aspect of JBioWH can prove to be very useful for complex queries that cannot be easily answered by SQL, for instance biological networks like drug-pathway as discussed in [28]. A more detailed comparison could not be made because many of the URLs to JBioWH are no longer valid.

*E-utilities* The E-utilities provide rich functionality in terms of data retrieval and cross-referencing, in particular when building data pipelines by merging successive E-utility calls. However, the E-utilities are primarily designed for NCBI resources. Thus, data from non-NCBI resources like Pfam and GO cannot be retrieved and cross-referenced. Also, E-utility applications are written in Perl, so the relationships between the different biological entities are not easily represented in the resulting dataset. Conversely, TAGOPSIN is written in Java to implement the concept of object-oriented biology, and it assembles annotated data from different sources into a single relational database.

*UniProtKB* UniProtKB is the most popular retrieval and cross-referencing tool for proteins and their annotations. Its ‘Retrieve/ID mapping’ tool allows a user to enter an identifier or a list of identifiers to perform pairwise cross-references with a multitude of other databases. This tool returns the identifier of the cross-referenced database. For example, in the case of RefSeq, when cross-referencing a given UniProt AC, ‘Retrieve/ID mapping’ provides only the RefSeq identifier. A user will then need to query NCBI RefSeq/GenBank to retrieve the complete set of annotation, which in the end amounts to a multi-database query.

Moreover, UniProtKB’s advanced search helps a user to carry out specific queries using logical operators to combine various search terms. We compared the output of the ‘Advanced Search’ functionality with that of TAGOPSIN for each one of the organisms listed in Table 1. We filtered our search to include only reviewed entries of UniProtKB (i.e. the Swiss-Prot subset) and entries that have a RefSeq cross-reference. The results are shown in Table 2.

**Table 2:** Comparison of the output between UniProtKB and TAGOPSIN

	No of Swiss-Prot entries returned by	
	UniProtKB	TAGOPSIN
*H. sapiens*	18 991	17 261
*A. thaliana*	15 669	15 383
*S. cerevisiae*	5 915	5 876
*E. coli*	22 218	17 777
*Streptococcus*	11 414	9 037
Human coronavirus	22	21
Human papillomavirus	128	127

UniProtKB entries (updated September 2020) were filtered using the ’Advanced Search’ functionality to include only reviewed entries having a RefSeq cross-reference for each one of the organisms indicated. Numbers for TAGOPSIN are from Table 1

Given that TAGOPSIN retrieves Swiss-Prot data from UniProt FTP server, one would have expected equal numbers of proteins returned by TAGOPSIN and by UniProtKB, though in some cases they are more or less the same (e.g. *A. thaliana, S. cerevisiae* in Table 2). The difference can be explained in terms of the relative date of update of information accessible via FTP or HTTP. Nonetheless, we argue that the added value of TAGOPSIN is the coding sequence data from GenBank, which we could not find on UniProtKB, and again they can be extremely useful in the context of comparative genomics.

*bioDBnet* Similar to UniProtKB, *db2db* of bioDBnet web server enables conversions of identifiers from one database to the other. Notwithstanding that a wide range of databases are represented and it is possible to limit the search to a given taxon, the output is the ID or a list of IDs of the cross-referenced database. Hence, much the same as UniProtKB, a user will still need to query the target database to get additional biological information.

Other tools within bioDBnet that could be relevant here are *dbWalk* and *dbAnnot*. They also map an ID onto a target database’s ID, can limit their search to a particular taxon, and in the case of *dbAnnot*, it provides annotations from the target database. We believe though that it will be difficult to address explicit queries which the versatility of SQL can help answer. Thanks to this versatility, TAGOPSIN provides a tailor-made resource for complex and specific queries regarding a particular taxon. Such queries include for example those that match a given keyword in the annotation or those that refine the result set according to some quantitative attribute.

*EBI SIFTS* SIFTS provides a comprehensive residue-level mapping between PDB structures and other biological databases of function, domain, taxonomy, and genome annotation. The mappings are available for download as CSV or TSV files. However, SIFTS gives only the identifier of the cross-referenced database. Besides, mapping is restricted to database entries with an experimentally solved 3D structure. Therefore, a complete dataset for a specific organism, irrespective of the availability of its proteins’ 3D structures, cannot be obtained using SIFTS.

*Bio4j, Neo4j* Graph databases such as Bio4j and Neo4j are used mainly to model molecular interactions and pathways. Neo4j in particular was used in a couple of previous studies to represent molecular interaction data namely PPI, drug-target, gene-disease, transcription factor-target gene etc. [32, 33]. We also understand that Neo4j is not a data retrieval or cross-referencing tool. This is confirmed by Yoon et al. and Lysenko et al. who “collected diverse biological network information from the web” [32, 33]. Conversely, data retrieval and cross-referencing are automated in TAGOPSIN, though its current scope does not cover biological network data. Furthermore, an interesting built-in feature of TAGOPSIN is that it caters for the data file format of each of its source databases. Neo4j however requires that the data be in CSV format only which can be a bottleneck for database integration.

*WikiGenomes* The WikiGenomes web application facilitates curation and use of genomic data for a given organism [31]. Similar to TAGOPSIN, WikiGenomes allows search and retrieval of biological data by organism. It uses the Wikidata data model, which is comparable to TAGOPSIN’s data model (Fig. 1 and Additional file 2). Nevertheless, even though WikiGenomes currently supports 120 NCBI prokaryotic reference genomes [31], it does not provide data for eukaryotes and viruses. For those prokaryotes where an output is provided, the genome browser displays gene and operon tracks. In addition, annotations from cross-referenced databases like GO, UniProt and NCBI RefSeq are hyperlinked to their respective database entries. Another major difference with TAGOPSIN is the editable and queryable graph database which defines Wikidata [31]. Here, we chose a relational database to design TAGOPSIN’s data warehouse.

On the whole, compared to the above-mentioned tools, the main strength of TAGOPSIN is that it builds an organism-centred data warehouse system from input at the command line. TAGOPSIN collates curated select data from its source databases so as to homogenise dataset preparation and lay the foundation for a broad range of investigations.

### Merits of the program

TAGOPSIN will be useful to evolutionary biologists, bioinformaticians, molecular biologists and structural biologists. In general, any scientist working on comparative genomics will find the tool very useful. We also illustrate the merits of the program with three case studies as outlined below.

#### Case study 1: molecular evolutionary analysis

In many molecular evolutionary and other comparative genomics studies, the datasets are very often retrieved manually as per each researcher’s methodology. For instance, in their evolutionary analysis of genome expansion and pathogenicity in *E. coli*, Bohlin et al. obtained from NCBI GenBank 53 fully sequenced *E. coli* genomes, with their annotated coding genes and corresponding proteins. Interestingly, all but 3 of the 53 genomes are retrieved by TAGOPSIN and organised in PostgreSQL. The missing 3 genomes are now obsolete. Moreover, while the names of the strains are the same, their genome ACs are different, due to the fact that TAGOPSIN extracts only curated, non-redundant data from GenBank. Further, to study the molecular evolution of the core genome, an all-against-all protein BLAST to obtain the core genome followed by gene-wise multiple alignments to compute values of *d*N and *d*S were performed [34]. These steps may involve getting protein IDs, or amino acid and nucleotide sequences. By collating all this information for the *E. coli* strains used in [34], we argue that TAGOPSIN can facilitate dataset preparation.

#### Case study 2: comparative genomics and phylogenetic analysis

Similarly, comparative genomics was carried out to infer the evolutionary relationship between the food-borne Shiga toxin-producing *E. coli* (STEC) O157:H7 strain NADC 6564 and other STEC O157 and non-O157 strains [35]. In the process of comparing the core genomes and estimating a phylogenetic tree, the authors downloaded from NCBI FASTA files containing the set of genes and their corresponding nucleotide sequences for the 40 genomes being compared. They also downloaded amino acid sequences of specific products for phylogenetic analysis. TAGOPSIN enables the retrieval of these data at once and organises them in a single data warehouse for ease of data manipulation. The *E. coli* dataset retrieved by TAGOPSIN included NCBI GenBank data for all 41 strains used by the authors, although 3 strains are referenced with a different RefSeq genome AC.

#### Case study 3: analysis of 3D structures in molecular docking and drug discovery

Latek et al. elucidated the potential binding mode and mechanism of action of human VPAC1 (vasoactive intestinal peptide receptor 1) antagonists by means of a workflow that included molecular docking and molecular dynamics simulations. VPAC1 has been selected for therapy of neurodegenerative disorder and inflammatory diseases, among others. Because there is no experimentally determined structure of VPAC1 in PDB, the authors used as template eight different PDB structures of closely-related receptors to reconstruct the extracellular and transmembrane domains of VPAC1. This model was then used for protein-ligand docking [36]. We show that all eight PDB entries form part of TAGOPSIN’s dataset for *H. sapiens* (Additional file 3).

### Limitations and known issues

A limitation of TAGOPSIN is its heavy dependence on Internet bandwidth. With a slow Internet connection, Wget may not always retrieve all available data from NCBI Nucleotide via E-utilities, thus resulting in an incomplete dataset. As a workaround, TAGOPSIN iteratively tries to download any missing file until the correct number of files has been downloaded.

For very large datasets, the Java Virtual Machine can run out of memory owing to the Java heap space. Here, it was observed for the *E. coli* dataset when mapping RefSeq protein IDs onto UniProt ACs. We got round the issue by adding extra options to the standard command in order to increase the heap size. These options are detailed in the README file available on the GitHub project homepage.

### Future directions

In view of making TAGOPSIN relevant to more focused studies, one of the developments will be to include other specialized databases within the scope of the program, such as the Kyoto Encyclopedia of Genes and Genomes (KEGG) [37].

## Conclusion

Many biological research questions can only be answered by combining information from a number of sources. Indeed, there is a need to search several databases and relying on only one is not sufficient. TAGOPSIN circumvents these limitations by using a standardised data model and a multi-database approach to efficiently assemble useful data from seven popular biological databases in a single repository. TAGOPSIN integrates these databases into a data warehouse that gathers the raw material for addressing a variety of research problems.

## Availability and requirements

- **Project name**: TAGOPSIN
- **Project home page**: https://github.com/ebundhoo/TAGOPSIN
- **Operating system(s)**: Unix-based (e.g. Linux, macOS)
- **Programming language**: Java 8
- **Other requirements**: PostgreSQL 9.6.5 or higher, GNU Wget
- **License**: GNU General Public License 3.0
- **Any restrictions to use by non-academics**: As per terms and conditions of license

## Supplementary information

**Additional file 1**. List of* Streptococcus* species and their corresponding genome ACs and CDS counts retrieved by TAGOPSIN.

**Additional file 2**: Extended ERD illustrating the data model of TAGOPSIN.

**Additional file 3**. PDB dataset retrieved by TAGOPSIN for case study 3 [36].

## Data Availability

Source code, binary file and documentation are available at https://github.com/ebundhoo/TAGOPSIN.

## Abbreviations

3D: Three-dimensional
AA: Amino acid
AC: Accession
BLAST: Basic local alignment search tool
CDS: Coding sequence
CPU: Central processing unit
CSV: Comma-separated values
DBVS: Docking-based virtual screening
DNA: Deoxyribonucleic acid
EBI SIFTS: European Bioinformatics Institute Structure Integration with Function, Taxonomy and Sequences
ERD: Entity-relationship diagram
FTP: File transfer protocol
GHz: Gigahertz
GiB: Gibibyte
GO: Gene Ontology
HTTP: Hypertext transfer protocol
ID: Identifier
JAR: Java archive
KB: Knowledgebase
NCBI: National Center for Biotechnology Information
NT: Nucleotide
PDB: Protein Data Bank
PPI: Protein-protein interaction
RAM: Random-access memory
RCSB PDB: Research Collaboratory for Structural Bioinformatics Protein Data Bank
SQL: Structured query language
STEC: Shiga toxin-producing *E. coli*
TSV: Tab-separated values
URL: Universal resource locator
VPAC1: Vasoactive intestinal peptide receptor 1

## Footnotes

1 https://biodbnet-abcc.ncifcrf.gov.

2 http://biowarehouse.ai.sri.com/EcoliHouseOverview.html.
