# Supplementary figures and images for: TAGOPSIN: collating taxa-specific gene and protein functional and structural information
Source: BMC Bioinformatics. 2021 Oct 23;22:517. doi: 10.1186/s12859-021-04429-5 (PMC8541804; doi:10.1186/s12859-021-04429-5)

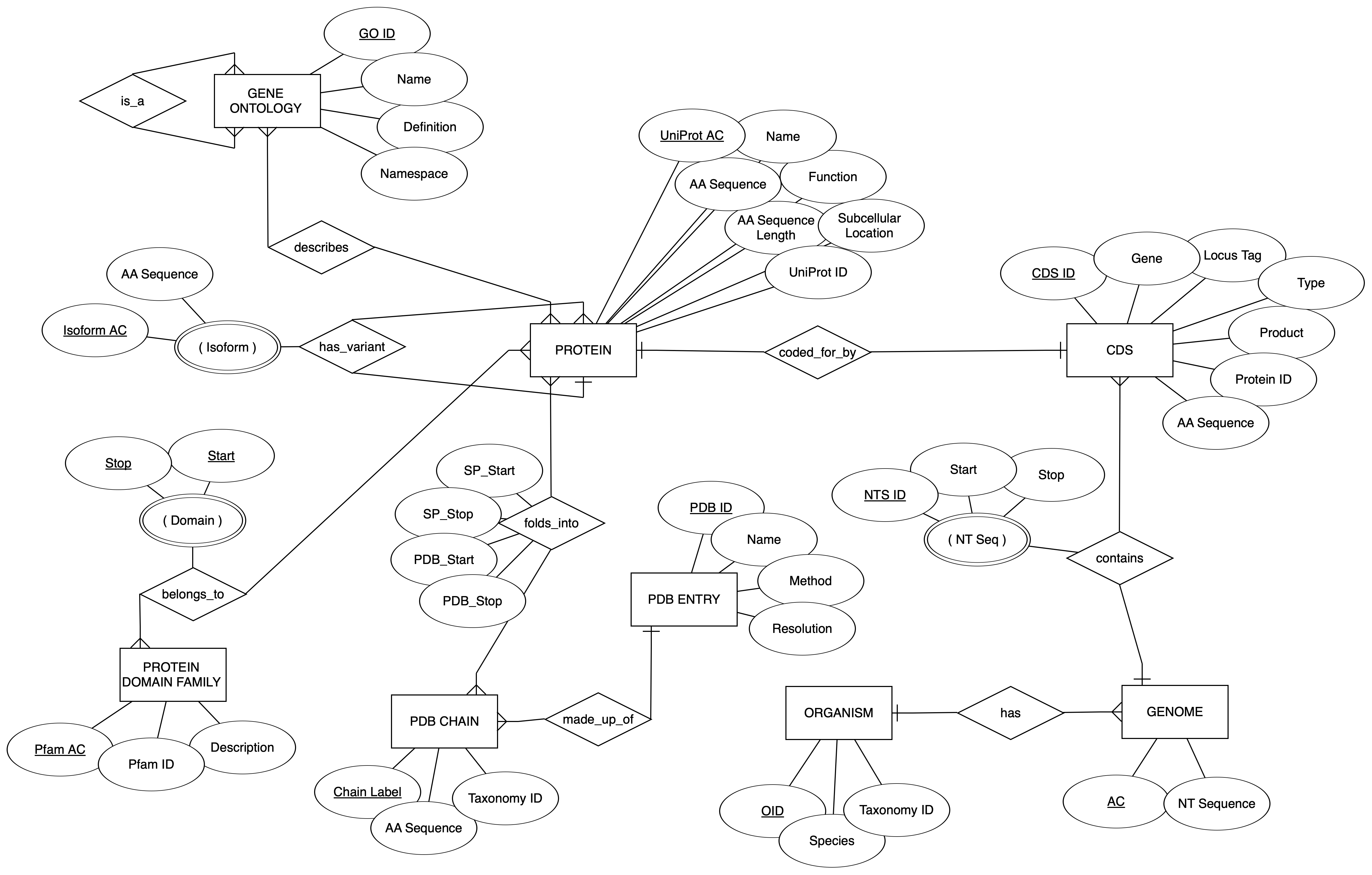

Supplement: Supplementary file 2 — Additional file 2: Extended ERD illustrating the data model of TAGOPSIN. [file 12859_2021_4429_MOESM2_ESM.png]
